# Factors associated with different forms of alcohol use behaviors among college students in Bhutan: a cross-sectional study

**DOI:** 10.1186/s13011-020-00315-0

**Published:** 2020-09-14

**Authors:** Tandin Dorji, Peeradone Srichan, Tawatchai Apidechkul, Rachanee Sunsern, Wipob Suttana

**Affiliations:** 1 School of Health Science, Mae Fah Luang University, Chiang Rai, Thailand; 2 District Health Sector, District Administration, Trashigang, Bhutan; 3 Center of Excellence for the Hill tribe Health Research, Mae Fah Luang University, Chiang Rai, Thailand

**Keywords:** Alcohol use, Behaviors, College student, Prevalence

## Abstract

**Background:**

Alcohol use has impacts on several dimensions of health, including physical health and mental health, particularly in college-age populations. Therefore, this study aimed to estimate the prevalence and to determine factors associated with alcohol use behaviors among college students in Bhutan.

**Methods:**

A cross-sectional design was applied to collect data from the participants. A simple random method was used to select the participants from the lists of students who were attending the four selected colleges in Bhutan in the academic year of 2019. A questionnaire was developed, and validity and reliability were verified before use. Descriptive statistics were applied to describe the general characteristics of participants, while logistic regression was used to detect the associations between variables at the significance level of α = 0.05.

**Results:**

A total of 432 college students were recruited into the study, of whom 62.0% were females. The average age was 20.0 years, 40.7% were the third-year students, and 79.9% lived in college dormitories. The prevalence of current drinking was 51.6% and that of binge drinking was 19.4%. After controlling for all potential confounder factors, three variables were found to be associated with current drinking: students who had low income were more likely to be current drinkers than those who had high income (AOR = 2.59, 95% CI = 1.29–5.21); students who did not use tobacco were more likely to be current drinkers than those students who used tobacco (AOR = 6.99, 95% CI = 2.90–16.81); and students who had close friends who did not use alcohol were more likely to be current drinkers than those who had close friends who used alcohol (AOR = 5.14, 95% CI = 3.04–8.69). Four factors were found to be associated with binge drinking after controlling for all possible confounder factors: students who had high income were more likely to be binge drinkers than those who had low income (AOR = 3.03, 95% CI = 1.72–5.31); students who used tobacco were more likely to be binge drinkers than those students who did not use tobacco (AOR = 2.28, 95% CI = 1.35–3.87); students whose parents used alcohol were more likely to be binge drinkers than those students whose parents did not use alcohol (AOR = 1.75, 95% CI = 1.02–3.01); and students whose close friends used alcohol were more likely to be binge drinkers than those who had close friends who did not use alcohol (AOR = 2.26, 95% CI = 1.05–4.87).

**Conclusions:**

There is a high prevalence in alcohol use among the college students in Bhutan. Effective health promotion programs should be implemented by focusing on reducing the alcohol consumption among college students.

## Background

Alcohol has been widely used in many cultures in different populations and different countries [1]. The impacts of alcohol use are clearly demonstrated in the development of many human diseases along with social and economic losses in societies [2]. Alcohol use is linked to more than 200 health problems, including alcohol dependence [3], liver cirrhosis [4], cancers [5] cardiovascular diseases [6], injuries due to violence [7], and road traffic accidents [8]. Significantly, the alcohol use-related burden is relatively affecting younger age groups, which can reduce their ability to learn and contribute to the socio-economic development of their nation in the future.

In 2020, the World Health organization (WHO) reported that 3 million deaths each year resulted from alcohol use, accounting for 5.3% of all deaths globally, and 13.5% of deaths among people aged 20–39 years are alcohol-attributed [9]. Approximately 5.1% of global burden and injury is attributed to alcohol use and measured in disability-adjusted life years (DALYs) [9]. Moreover, many severe infectious diseases are related to alcohol use, such as tuberculosis [10] and human immunodeficiency syndrome (AIDS) among young people [11, 12].

Alcohol use is classified into several forms dominating in different groups of people, such as binge drinking and current drinking. The Centers for Disease Control and Prevention (CDC) defines that binge drinking is a pattern of drinking that brings a person’s blood alcohol concentration (BAC) to 0.08 g percent or above, which can occur when a man drinks 5 or more drinks or a woman drinks 4 or more drinks in approximately 2 h [13]. Two forms of drinking behaviors that greatly impact drinkers, particularly young adults or college students, are binge drinking and current drinking behaviors.

Young adults, particularly those who are attending college or university, are the most vulnerable to alcohol use [9]. The WHO also reported that 13.5% of deaths among young adults are alcohol-attributable [9]. In 2017, the prevalence rates of binge drinking were 34.8% among college students in the United States [14], 51.6% in Brazil [15], 22.2% in South Africa [16], and 34.1% in Uganda [17]. In Asia, the prevalence rates of binge drinking among college students were 23.5% in China [18], 67.9% in Japan [19], and 39.1% in Thailand [20]. The prevalence rates of current drinking among the young adults were different in different countries: 9.7% in Iraq [21], 13.77% in Iran [22], 17.6% in Romania [23], 31.1% in Nigeria [24], 20.3% in Nepal [25], 39.4% in India [26], and 60% in males and < 5% among females were currently use among the Vietnam people [27].

There are several impacts of alcohol use among young adults, particularly those who are attending university or college, such as reduced learning capability [28], deprived coping skills [29], and condensed social skills [30]. Several studies [15, 21, 24] have reported that different characteristics such as sex, age, living away from home, parental education, household income, and tobacco use, contribute to the prevalence of alcohol use. However, there is limited information on the magnitude and factors associated with alcohol use behaviors among university or college students in Bhutan, which could be used for appropriate public policy development and the eventual deployment of effective public health interventions.

## Methods

### Study design

A cross-sectional study was used to estimate the prevalence and to determine the factors associated with different forms of alcohol use behaviors among college students in Bhutan.

### Study setting

This study was conducted in constituent colleges under the Royal University of Bhutan. In 2019, there were 10 constituent colleges and 2 affiliated colleges under the Royal University of Bhutan [31]. Out of 10 constituent colleges, 4 colleges were included as a study setting in this study. The four colleges had the highest number of students among other colleges under the Royal University of Bhutan in the same year.

### Study population

College students attending the four selected colleagues in the 2019 academic year were the study population.

### Study sample and sample size

Students who could provide all essential information in the questionnaires met the eligibility criteria for the study. According to the annual reports of the Royal University of Bhutan, there were 5909 students in 4 selected colleges on the date of the study in 2019. After using the standard formula for sample size, a margin of error of 0.05 (5.0%) around a prevalence of 0.5 (50.0%) [32], at least 360 participants were needed for the analysis. After adding 20.0% to account for any errors or missing data throughout the study process, a total of 432 participants were determined to be required for the study.

### Research instruments

The structured questionnaire was developed from the literature review of various sources of information. Questionnaires were developed in English that consisted of four sections. In the first part, 7 questions were used to collect sociodemographic information of the participants, including age, sex, year of study, place of residence, and household income of the participant. In the second part, 4 questions were used to collect information on alcohol use behaviors; (1) “Have you ever consumed at least one standard drink of any alcohol drink in your life time?”, (2) “Have you consumed at least one standard drink of any alcohol drink in past 30 days?”, (3) “During the 30 days, what was the largest number of standard alcoholic drinks you had on a single occasion, counting all types of alcoholic drinks together?”, and (4) “Did you drink of ≥5 standard drinks of alcohol for male or ≥ 4 standard for female? .

Based on question no. (1) was used for identifying life time drinking, question no (2) was used for identifying current drinking behavior, and question no. (3) and (4) were used for identifying in binge drinking behaviors of the participants.

In the third part, 2 questions were used to collect information about substance use behaviors, such as “Do you use any smoke or smokeless tobacco products (such as cigarettes, snuff, chewing tobacco, cigars, etc.)?” and “Do you currently use any drugs (such as tablets and marijuana, etc.)?” In the last part, 3 questions were used to collect information about parental education, parental alcohol use and close friends’ alcohol use.

The validity was tested by using the Item Objective Congruence Index (IOC) method [33] by three experts in the field, consisting of two public health officers and one medical doctor. Those questions that scored less than 0.5 were deleted from the questionnaire, while those that scored 0.5–0.7 were revised as per the comments from the committee before they were incorporated into the questionnaire set. Questions that scored greater than 0.7 were pulled into the questionnaire set without revision.

A pilot was executed among 15 samples who were students attending from four selected colleges to test for the appropriateness of questions and the order of the questions in the questionnaire. The pilot was conducted 6–7 August 2019 for two times.

### Data gathering procedure

Four of 10 Constituent Colleges under the Royal University of Bhutan were selected for the study settings. In 2019, a total of 5909 students were studying in those four colleges. After getting the student lists from all four universities, they were placed in the same file by continuous names, and a random method was then used to select from the list, and 432 students were chosen. All selected students were contacted and appointed ahead. Prior to the data collection day, students’ representatives were briefed about the purpose and objective, the content of the questionnaire and the data collection process. On the date of data collection, all participants were obtained informed consent form on voluntary basis before starting the study. The questionnaires were mostly completed using a self-administered method after informed consent was obtained from the participants. The questionnaires were returned to the researchers upon completion and were checked for missing answers before the data collection process was considered complete.

### Statistical analysis

Data were coded, entered and rechecked using SPSS version 20.0 (Chicago, IL). After cleaning for errors and missing values, coding, sorting and recording were carried out. Descriptive statistics in the continuous data are presented as frequency, mean, maximum, minimum and standard deviation to explain the distribution of characteristics of the participants. The categorical data are presented as percentage. Logistic regression was used to test the associations between variables at a significance level α = 0.05.

## Results

A total of 432 participants from four colleges under the Royal University of Bhutan were recruited into this study. More than half of the participants (55.8%) were under the age of 21–24 years (mean = 20.08, SD = 1.6). The majority of the participants were females (62.0%), were in their third year of college (40.7%), lived in the college dormitory (79.9%) and had low household income (75.0%) (Table 1).

**Table 1:** General characteristics of the participants

Characteristics	n	%
**Total**	**432**	**100**
**Age** (years)		
17–20	186	43.1
≥ 21	246	56.9
Mean = 20.08, SD = 1.57, min = 17 max = 29		
**Sex**		
Male	164	38.0
Female	268	62.0
**Year of study**		
First year	106	24.5
Second year	150	34.7
Third year	176	40.7
**Residence**		
Living with parents	79	18.3
College dormitory	345	79.9
Apartments outside college	8	1.8
**Household income** (Ngultrum)		
Low income (< 164,829)	324	75.0
High income (≥164, 829)	108	25.0
**Tobacco use** (Cigarettes, snuff, chewing tobacco, cigars)		
Yes	130	30.1
No	302	69.9
**Drug use** (Tablets and marijuana)		
Yes	30	6.9
No	402	93.1
**Age of initiation of drinking**		
< 18	200	46.3
≥ 18	232	53.7
**Father’s education**		
No formal education	178	41.2
Primary education	80	18.5
Secondary education	81	18.7
Higher education	93	21.5
**Mother’s education**		
No formal education	290	67.1
Primary education	71	16.4
Secondary education	47	10.9
Higher education	24	5.6
**Parent’s alcohol use**		
Yes	247	57.2
No	185	42.8
**Having close friend who use alcohol**		
Yes	329	76.2
No	103	23.8

Regarding substance use, 30.1% used tobacco (smoke or smokeless), while only 6.9% used drugs such as tablets and marijuana. Regarding parents’ education, 41.2% responded that their father had no formal education, while 18.5% had attended primary education. In terms of their mother’s education, 67.1% had no formal education, and only 5.6% had attended higher education. More than half (57.1%) reported that their parents used alcohol, and 76.2% responded as having close friends who used alcohol (Table 1).

The prevalence of current drinkers was 51.6%, the prevalence of binge drinking was 19.4%, and the prevalence of lifetime alcohol use was 78.0%.

In the univariable analysis in detecting the factors associated with current drinking, 3 variables were found to be associated: household income, tobacco use and having close friends who use alcohol. However, after controlling for all potential confounder factors, three factors were to be associated with current drinkers. Participants who had low household income were 2.59 times more likely to be current drinkers than those who had high income (95% CI = 1.29–5.21). Participants who did not use tobacco were 6.99 times more likely to be current drinkers than those who used tobacco (95% CI = 2.90–16.81). Participants whose had close friends who did not use alcohol were 5.14 times more likely to be current drinkers (95% CI = 3.04–8.69) (Table 2).

**Table 2:** Univariable and multivariable analyses of factors associated with different forms of alcohol use among the college students

Factor	Current drinking						Binge drinking					
	Univariable analysis			Multivariable analysis			Univariable analysis			Multivariable analysis		
	OR	95%CI	***p***-value	AOR	95%CI	***p***-value	OR	95%CI	***p***-value	AOR	95%CI	***p***-value
**Age** (years)												
≤ 20	1.00						1.00					
> 20	0.84	0.53–0.32	0.444				0.95	0.59–1.54	0.838			
**Sex**												
Male	1.00						1.00					
Female	1.23	0.77–1.95	0.389				0.69	0.42–1.10	0.687			
**Year of study**												
First Year	1.00						1.00					
Second year	1.21	0.66–2.21	0.540				1.07	0.57–2.02	0.822			
Third year	0.93	0.51–1.69	0.825				1.03	0.56–1.90	0.926			
**Residence**												
Living with parents	1.00						1.00					
College dormitory	1.39	0.76–2.52	0.285				0.71	0.39–1.27	0.254			
**Household income (**Ngultrum)												
Low income (< 164,829)	1.79	1.05–3.04	0.032*	2.59	1.29–5.21	0.007*	1.00			1.00		
High income (≥164,829)	1.00			1.00			3.07	1.86–5.08	< 0.001*	3.03	1.72–5.31	< 0.001*
**Tobacco use**												
Yes	1.00			1.00			2.60	1.59–4.25	< 0.001*	2.28	1.35–3.87	0.002*
No	2.65	1.59–4.24	< 0.001*	6.99	2.90–16.81	< 0.001*	1.00			1.00		
**Drug use**												
Yes	1.24	0.54–2.84	0.604				1.87	0.82–4.24	0.135			
No	1.00						1.00					
**Father’s education**												
No formal education	1.00						1.00					
Primary education	1.11	0.61–2.01	0.741				1.28	0.65–2.56	0.469			
Secondary education	0.83	0.40–1.69	0.602				0.81	0.38–1.71	0.576			
Higher education	1.09	0.54–2.20	0.799				2.21	1.22–4.01	0.009*			
**Mother’s education**												
No formal education	1.00						1.00					
Primary education	0.61	0.32–0.18	0.142				1.27	0.65–2.46	0.48			
Secondary education	1.23	0.56–2.28	0.736				2.19	1.09–4.41	0.028*			
Higher education	0.79	0.31–2.00	0.619				3.10	1.28–7.50	0.012*			
**Parents drinking**												
Yes	0.82	0.52–1.29	0.395				1.76	1.06–2.91	0.029*	1.75	1.02–3.01	(0.042*)
No	1.00						1.00			1.00		
Having close friend who use alcohol												
Yes	1.00			1.00			3.08	1.48–6.40	0.003*	2.26	1.05–4.87	(0.038*)
No	3.26	1.78–5.98	< 0.001*	5.14	3.04–8.69	< 0.001*	1.00			1.00		

*Significant level at α = 0.0

In the univariable analysis for detecting the factors associated with binge drinking among college students, 6 variables were found to be associated with binge drinking; household income, father’s education, mother’s education, tobacco use, parents’ drinking, and close friends who used alcohol. However, after controlling for all potential confounder factors, four factors remained to be associated with binge drinking; high household income, smoking, parents’ drinking, and having close friends who used alcohol. Participants who had high household income were 3.03 times more likely to binge drink than those who had low household income (95% CI = 1.72–5.31). Participants who smoked were 2.28 times more likely to binge drink than those who did not smoke (95% CI = 1.35–3.87). Participants whose parents used alcohol were 1.75 times more likely to binge drink than those parents who did not use alcohol (95% CI = 1.02–3.01). Participants whose close friends used alcohol were 2.26 times more likely to binge drink than who did not (95% CI = 1.05–4.87) (Table 2).

## Discussion

Among the college students in Bhutan, a large proportion were reported to have lifetime alcohol use (78.0%), current alcohol drinking (51.6%), and experience in binge drinking (19.4%). Almost half (64.3%) of the college students reported that they initiated the use of alcohol before 18 years, a large proportion (76.2%) had close friends who used alcohol, and more than half (57.2%) reported having parents who used alcohol. Several factors were found to be associated with the current use of alcohol and binge drinking: family income, tobacco use, and having close friends who used alcohol. However, some detected factors showed an opposite association for current drinking and binge drinking; for example, those who had low family income had a greater chance to be current alcohol users, while the high family income students had a greater chance of engaging in binge drinking. Similarly, those who did not use tobacco had a greater chance to be current alcohol users than those who used tobacco, while in the binge drinking behaviors, the opposite association was found.

The findings of the study presented the high prevalence of alcohol use among the college students in various forms, which was not too much over the estimation of the research, which was much higher than that of the adult population at 30.9% regarding current use [34]. Based on the culture and lifestyle of people in Bhutan, these behaviors are very much related to religion. Alcohol is also always used in tantric rituals as a symbol of wisdom, which is made in two forms: during religious rites and during ceremonies for blessings on any new ventures. Alcohol use is common for both males and females, and nearly everyone is introduced to the use of alcohol at an early age [35]. Moreover, accessibility to alcohol was one important factor in the introduction of alcohol use for adolescents who do not have any social restriction on the use alcohol among both males and females, as it is widely accessible in Bhutan. The production and consumption of homebrews such as ara (distilled from grains), bangchang (fermented and extracted from grains), singchang (extracted from grains), tongba, and chang-kyod were found to constitute the main drinks in Bhutan according to the Bhutan Living Standard Survey [36].

In our study, it was found that those students who were from low-income families were likely to be current alcohol users than those who lived-in high-income families. However, the opposite direction was presented for binge drinking, as those who lived in a high-income family were more likely to experience binge drinking than those who lived in a low-income family. A study in Finland [37] reported that among university students, both males and females who had a high income were significantly more likely to use alcohol in all forms than those living in a low-income family. In contrast, Katikireddi, et al. [38] demonstrated that those who lived in a low socioeconomic status were consistently associated with strikingly increased alcohol-attributable harms, including binge drinking. A study in Southwest England reported that students or adolescents from higher-income households were at greater risk alcohol use and some types of adolescent alcohol problems than those students or adolescents from low-income households [39].

Regarding tobacco use, it was found that those college students in Bhutan who did not use tobacco were more likely to be current alcohol users than those who did not use tobacco. However, while looking into the association between tobacco use and binge drinking, it was found that those who used tobacco were more likely to experience binge drinking than those who did not use it. A Norwegian study [40] reported that smoking or tobacco use was associated with alcohol use, particularly among men. Conversely, a study in Namibia [41] reported that both males and females who used tobacco were associated with alcohol use behaviors, but females had greater odds than males. In the context of Bhutan, males favor to have smoking behavior than female [35]. However, with a greater proportion of female participants (62.0%) in the study, then it’s possible to see unusual odds of the relationship between smoking and alcohol, but it would be common in Bhutan.

Having close friends who used alcohol was associated in a positive direction with binge drinking. Thus, college students who had close friends who used alcohol were more likely to experience binge drinking than those who did not have close friends who used alcohol. This profile had the opposite association with current alcohol use. Those college students who had close friends who did not use alcohol were more likely to be current alcohol users than those who had close friends who used alcohol. A study in the Unites States [42] clearly reported that those adolescents who had friends or peers who used alcohol were more likely to use alcohol and to experience binge drinking than those who did not have friends or peers who did not use alcohol. However, a longitudinal analysis indicated that friends’ alcohol use was not a good predictor of use among the teenagers [43]. However, a study in Australia [44] reported that alcohol was a friendship-making tool among young adults and that those young adults who had close friends who used alcohol also tended to use alcohol.

In this study, it was found that those participants whose parents used alcohol were more likely to experience binge drinking compared to those whose parents did not use alcohol. These findings were supported by a study conducted in Europe [45], which found that those teenagers who had parents who used alcohol tended to use alcohol compared to the opposite group significantly. A prospective cohort study [46] also found that parents’ alcohol use impacted alcohol use behaviors in their children in all forms, including binge drinking and alcohol-related harms. Moreover, a path model analysis [44] revealed a strong association between parents’ alcohol use and alcohol use among adolescents, which clearly demonstrated that children who lived in families whose parents used alcohol were more likely to use alcohol than those who had parents did not use alcohol significantly.

In the study process, we used a validated questionnaire, which was developed under the circumstance of Bhutanese cultures and societal perception on alcohol use among the young adult population or university students. Its validity and reliability were also verified in samples who lived in Bhutan and had similar characteristics with the study samples before use. Therefore, the gathered data were mostly accurate and reflected the situation of the study population. Moreover, the data collection step was conducted and monitored by one of our research team who is Bhutanese and just graduated from his undergraduate program a few years ago. With the condition of not too many years’ age difference between the researcher and the study participants, the data obtained were clearly proof the objective of the study.

## Conclusion

Alcohol consumption among college students in Bhutan is highly prevalent and common in both sexes. However, the prevalence rates of alcohol use in different forms are different. Some profiles of students, including living environments, were associated with alcohol use, such as living in high- or low-income families, behavior of tobacco use, having close friends who used alcohol and parents’ behaviors in the use of alcohol. Based on the traditional pattern of the people in Bhutan, they usually start to use alcohol some years after birth, related to the cultures and lifestyles of the people. Alcohol is used for several occasions that are related to religion’s rituals that occur many times year-round. However, many impacts from the use of alcohol have been found, particularly in young people who are attending college or university. Thus, a public health initiative to reduce alcohol use among university students or to delay use until the end of the university life should be promoted for achieving the optimal effect on human resource production in the country.

## Data Availability

The raw data supporting these findings can be found in the supplementary file.

## Abbreviations

WHO: World Health Organization
DALYs: Disability-adjusted life years
AIDS: Acquired immune deficiency syndrome
CDC: Centers for Disease Control and Prevention
OR: Odd ratio
